# New and Old Key Players in Liver Cancer

**DOI:** 10.3390/ijms242417152

**Published:** 2023-12-05

**Authors:** Ángel M. Cuesta, Nerea Palao, Paloma Bragado, Alvaro Gutierrez-Uzquiza, Blanca Herrera, Aránzazu Sánchez, Almudena Porras

**Affiliations:** 1 Departamento de Bioquímica y Biología Molecular, Facultad de Farmacia, Universidad Complutense de Madrid (UCM), 28040 Madrid, Spain; angcuest@ucm.es (Á.M.C.); npalao@ucm.es (N.P.); pbragado@ucm.es (P.B.); alguuz@ucm.es (A.G.-U.); blancamh@ucm.es (B.H.); munozas@ucm.es (A.S.); 2 Instituto de Investigación Sanitaria del Hospital Clínico San Carlos (IdISSC), 28040 Madrid, Spain; 3 Centro de Investigación Biomédica en Red de Enfermedades Hepáticas y Digestivas (CIBEREHD-ISCIII), 28040 Madrid, Spain

**Keywords:** liver cancer, tumor microenvironment, platelets, HGF, MET, EGFR, TGF-β, C3G/RAPGEF1

## Abstract

Liver cancer represents a major health problem worldwide with growing incidence and high mortality, hepatocellular carcinoma (HCC) being the most frequent. Hepatocytes are likely the cellular origin of most HCCs through the accumulation of genetic alterations, although hepatic progenitor cells (HPCs) might also be candidates in specific cases, as discussed here. HCC usually develops in a context of chronic inflammation, fibrosis, and cirrhosis, although the role of fibrosis is controversial. The interplay between hepatocytes, immune cells and hepatic stellate cells is a key issue. This review summarizes critical aspects of the liver tumor microenvironment paying special attention to platelets as new key players, which exert both pro- and anti-tumor effects, determined by specific contexts and a tight regulation of platelet signaling. Additionally, the relevance of specific signaling pathways, mainly HGF/MET, EGFR and TGF-β is discussed. HGF and TGF-β are produced by different liver cells and platelets and regulate not only tumor cell fate but also HPCs, inflammation and fibrosis, these being key players in these processes. The role of C3G/RAPGEF1, required for the proper function of HGF/MET signaling in HCC and HPCs, is highlighted, due to its ability to promote HCC growth and, regulate HPC fate and platelet-mediated actions on liver cancer.

## 1. Introduction

Liver cancer (including hepatocarcinoma (HCC), cholangiocarcinoma (CCA), and combined hepatocellular-cholangiocarcinoma (cHCC-CCA)) represents a global health challenge, and its incidence is growing worldwide. HCC is the most common form of primary liver cancer accounting for around 90% of cases [1]. According to the World Health Organization (WHO), in 2020 liver cancer was the sixth most common cancer worldwide in incidence, accounting for 4.7% of cases, and the third most common in the number of cancer-related deaths, accounting for 8.3% of cases. However, there is a striking variation in HCC incidence rates across geographic regions. The highest incidence and mortality of HCC are observed in East Asia and Africa, but they are also increasing in Europe and the USA. Furthermore, HCC has a strong male predominance likely related to a clustering of risk factors and differences in sex hormones [2,3,4].

The etiology of HCC and the implicated signaling mechanisms need to be fully characterized. Liver cancer, specifically HCC, is usually associated with chronic inflammation, fibrosis and cirrhosis [1]. However, a new HCC subclass, not associated with inflammation, and enriched in *CTNNB1* gene mutations and PTK2/FAK1 overexpression has been described [5]. Fibrosis is a common manifestation of chronic liver disease (CLD) that can progress to cirrhosis, eventually leading to HCC development. Several risk factors, frequently associated with chronic liver inflammation, are involved in HCC development, including hepatitis viruses, carcinogens, alcohol intake, hereditary diseases, metabolic syndrome, and fatty liver disease [4,6,7]. Hepatitis B virus (HBV) infection is the most prominent risk factor for HCC, while the risk attributed to hepatitis C virus (HCV) infection has decreased thanks to antiviral treatments and the extended use of vaccines. In contrast, non-alcoholic fatty liver disease (NAFLD) and non-alcoholic steatohepatitis (NASH), a more severe form of NAFLD, are becoming the fastest growing causes of HCC, which are not associated with cirrhosis in most cases [8]. NAFLD and NASH are strongly connected with metabolic syndrome (a condition associated with type 2 diabetes, dyslipidemia, and hypertension) [9]. Therefore, in 2020, a group of international experts in the field proposed a change of the term from NAFLD to MAFLD (metabolic associated fatty liver disease) [10]. Moreover, in recent years, it has been considered that HCCs generated by MAFLD present specific characteristics [8], being more differentiated and having a higher inflammatory component.

HCC is the result of a complex long-term multistep process regulated by the interplay of several risk factors, and the severity of the underlying CLD. HCC results from the accumulation of somatic genetic and epigenomic alterations in the cells of origin. There is an average of 40–60 somatic mutations in coding regions, most of them in ‘passenger’ genes, but a few in cancer ‘driver’ genes, involved in activating key signaling pathways for hepatocarcinogenesis such as those implicated in telomere maintenance, cell cycle control, chromatin modification, and oxidative stress, with WNT–β-catenin, transforming growth factor-β (TGF-β) signaling and receptor tyrosine kinase (RTK) signaling cascades being relevant, among others [11,12,13,14].

Early-stage HCC lesions are small and can normally be cured by minimally invasive methods. Nevertheless, around 50% of the cases are diagnosed after developing symptomatic advanced-stage HCC. This significantly minimizes the possibility of treatment, and death is ensured after a few months [1,15]. Different options for first-, second- and subsequent line treatments are currently available, such as combinations of immunotherapy with tyrosine kinase inhibitors (TKIs) or antiangiogenic agents (e.g., vascular endothelial growth factor (VEGF) inhibitors) [16,17].

## 2. Liver Cancer Microenvironment

The cross-talk between hepatocytes and other cell populations, constituents of the liver microenvironment, such as hepatic stellate cells (HSCs), liver sinusoidal endothelial cells (LSECs), liver-resident macrophages Kupffer cells, KCs), other immune cells, and hepatic progenitor cells (HPCs), is involved in hepatocarcinogenesis in the context of CLD, and in metastasis generation in later stages [1,18]. In addition to this, the recruitment of immune cells and platelets to the liver during CLD also contributes to the hepatocarcinogenic process. The release of several growth factors, cytokines, chemokines, and extracellular matrix (ECM) proteins by liver cells and/or newly recruited cells, plays a major role in this process. Hence, this makes the pre-cancerous microenvironment of CLD to evolve towards a tumor microenvironment (TME) with immunosuppressive properties and altered angiogenesis, favoring the tumorigenic process. However, the TME is highly heterogeneous, which hinders its study within the tumor tissue in patients. Hence, recent works have also paid attention to the peritumor microenvironment (PME), a non-tumor tissue also involved in the occurrence (PME-O) and progression (PME-P) of HCC. Different proteome profiles have been identified in PME-O and PME-P, which are immune proteins’ key players in PME-O, while proteins involved in inflammation, angiogenesis and metabolism are relevant in PME-P [19].

It is also noteworthy to mention that despite having common characteristics, TME features are likely dependent on liver cancer etiology. The role and relevance of some major components of the liver microenvironment in HCC development and progression are further discussed below.

### 2.1. Fibrosis: Hepatic Stellate Cells and Cancer Associated Fibroblasts

As mentioned before, the majority of HCCs arise in a fibrotic or cirrhotic liver [20]. Fibrosis results from a chronic liver injury. The activation of HSCs and their further conversion into myofibroblasts (MFBs) are considered key steps in fibrosis development. However, MFBs can have different origins [21]. HSCs are the major source, but other cells such as portal fibroblasts, hepatocytes undergoing epithelial-mesenchymal-transition (EMT), fibrocytes (bone marrow-derived cells), or HPCs can also lead to MFBs [22].

**Figure 1:**
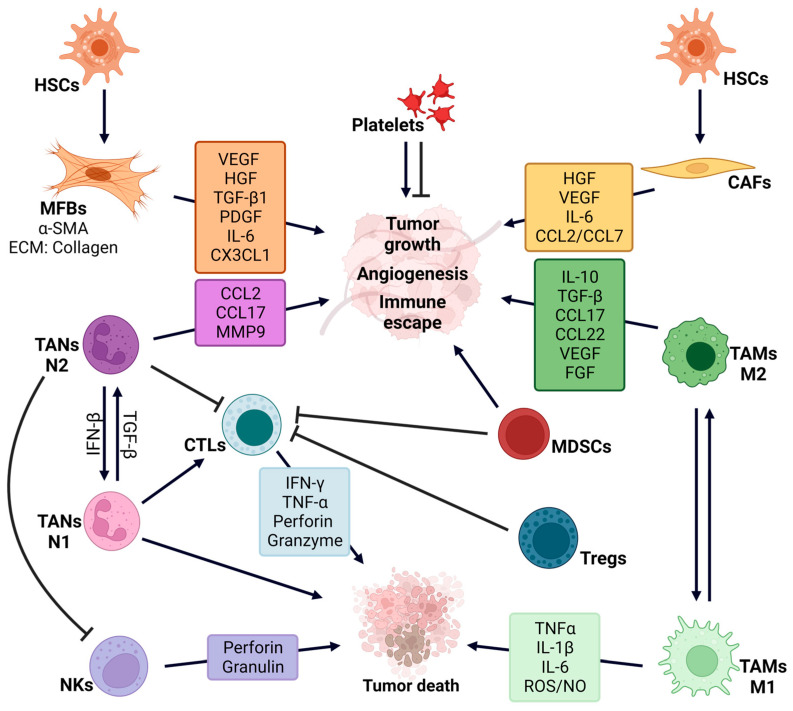
*Regulation of liver tumor development and progression by the tumor microenvironment***.** The role played by different constituents of liver tumor microenvironment is shown as well as the mediators of their actions (growth factors, cytokines, chemokines, and other molecules). Cell populations include: myofibroblasts (MFBs), cancer associated fibroblasts (CAFs), platelets and different populations of the immune system: cytotoxic T lymphocytes (CTLs), regulatory T lymphocytes (Tregs), M1 and M2 tumor associated macrophages (TAMs), myeloid derived suppressor cells (MDSCs), N1 and N2 tumor associated neutrophils (TANs) and natural killer cells (NKs). Created in BioRender.com.

In a normal liver, HSCs are non-proliferative cells that express glial fibrillary acidic protein (GFAP), desmin, vimentin, and platelet-derived growth factor receptor (PDGFR), among other markers [21]. However, upon chronic liver injury they are activated by growth factors mainly released by KCs, such as TGF-β1, considered their major activator, and cytokines such as tumor necrosis factor-α (TNF-α) and interleukin-1β (IL-1 β) [23,24]. After its activation and transdifferentiation into MFBs [25], they express α-smooth muscle actin (α-SMA) and ECM proteins, mainly collagen I and II [22]. In addition, activated HSCs also secrete TGF-β1 and chemokines such as chemokine (C-C motif) ligand 2 and ligand 5 (CCL2, CCL5) and chemokine (C-X-C motif) ligand 8-10 (CXCL8-10) [26] that regulate immune cells, fibrosis and hepatocarcinogenesis (Figure 1). Hence, during fibrosis development HSCs undergo dramatic transcriptomic changes with the sequential activation of inflammatory, migratory, and ECM–producing programs, as determined by single cell RNA sequencing (sc-RNAseq) [27]. Continuous activation of HSCs leads to a non-resolutive repair response evolving to fibrosis and cirrhosis.

Although fibrosis, cirrhosis and HCC are strongly associated, the casual relationship between fibrosis and HCC development is still unclear [28]. The use of different mouse models has led to controversial results. Some studies have shown an association between reduced fibrosis and less HCC risk [29,30]. Nevertheless, other studies did not find a correlation between fibrosis and HCC [31]. These discrepancies might be due to the existence of different subtypes of HSCs with specific functions. The analysis of human and mouse data revealed the existence of two main subtypes of HSCs in the fibrotic liver with opposed functions in hepatocarcinogenesis [32]. On one side, there are quiescent and cytokine-producing HSCs enriched in HGF (hepatocyte growth factor) that protect against hepatocyte cell death and HCC development and on the other side, activated myofibroblastic HSCs enriched in collagen I, which promote HCC development and increase ECM stiffness. An imbalance between these HSC types in favor of myofibroblastic HSCs during CLD favors HCC promotion. In relation to this, matrix stiffness could also play a key role. This is supported by recent studies indicating that a higher matrix stiffness promotes a cancer stem-like phenotype [33] and HCC progression [34]. More recent data derived from sc-RNAseq analyses support a more complex situation due to the existence of several subpopulations of HSCs in CLD [35]. Hence, activated HSCs are considered to be highly heterogenous and have been classified into distinct subpopulations: proliferative HSCs (pHSCs), inflammatory HSCs (iHSCs), intermediate activated vascular HSCs (vHSCs), contractile and migratory HSCs (cmHSCs), and fibrogenic myofibroblasts (myHSCs). However, the specific functions of each subpopulation in liver cancer as well as the regulatory signals remain unclear.

In the classic model, once liver cancer is generated, HSC activation in the peritumoral tissue contributes to HCC progression [20,36] through the secretion of growth factors, cytokines and chemokines such as VEGF, HGF, TGF-β1, PDGF, IL-6 and CX3CL1. These signals can act either directly on cancer cells inducing survival, proliferation and/or migration, or indirectly by enhancing angiogenesis and modifying the immune response to favor an immunosuppressive context. For example, KCs are stimulated by HSC-derived cytokines leading to the exhaustion of activated T cells [37,38], and the expansion of regulatory T cells (Tregs) or M2 macrophage polarization. An anti-inflammatory phenotype can also be induced by HSC-derived CX3CL1 through regulation of KCs and infiltrated monocytes [39,40]. Recently, through a sc-RNAseq analysis of human liver samples, new subpopulations of pro-fibrotic macrophages, endothelial cells, and PDGFRα+ collagen-producing mesenchymal cells which collaborate to promote fibrosis have been identified [41].

During HCC progression, HSCs and HSC-derived MFBs are believed to transform into cancer-associated fibroblasts (CAFs) [28], although CAFs could also be originated from cancer cells, endothelial cells, portal cells or mesenchymal bone marrow stem cells [42]. CAFs play a key role inhibiting immune cell response, promoting angiogenesis and HCC progression by secreting growth factors, cytokines and chemokines such as HGF, IL-6, VEGF, CCL2, or CCL7 [18,43,44,45]. However, the existence of different types of CAFs with distinct gene expression profiles and functions in hepatocarcinogenesis has recently been uncovered [35]. Among them, myofibroblastic CAFs, which produce collagen I, suppress tumor growth by mechanically restraining tumor spread, while inflammatory CAFs which secrete HGF, promote tumor growth [46]. Nevertheless, CAFs are heterogeneous and suffer dynamic changes during hepatocarcinogenesis that are likely dependent on the specific context.

Based on the above information, we can state that the classic association between fibrosis and liver cancer still stands. In this respect, there is a great interest in blocking transdifferentiation of HSCs into MFBs or, at least, to reduce it, as a potential therapy for cancer. However, recent published data support the idea of a more complex activity of HSCs and CAFs as they have different phenotypes and functions. This likely determines matrix stiffness and the secretion of specific growth factors, cytokines and chemokines, which leads to the activation or inhibition of tumor progression. In addition, HSCs and CAFs play an important role in the immune response as they can exert an immunosuppressive effect. Therefore, more efforts are required to understand all the complexity of fibrosis and its relationship with liver cancer.

### 2.2. Liver Endothelial Sinusoidal Cells

Liver sinusoidal endothelial cells (LSECs), mainly localized in the liver sinusoidal wall, have open fenestrations and lack a basement membrane to facilitate substance exchange from the sinusoidal space to the parenchyma and the space of Disse. Upon the alteration of KCs function, LSECs uptake foreign substances from the blood stream [42].

In response to liver injury, LSECs also contribute to induce HSCs proliferation by releasing VEGF [24]. In addition, they play a relevant role regulating the immune system and tumorigenesis [42], contributing to leukocyte recruitment into the injured liver [47].

### 2.3. Liver Immune Cells

The liver is an organ rich in immunocompetent cells [42,48,49]. Different immune cell populations are present in the liver under normal conditions, including KCs, natural killer (NK) cells, lymphocytes (from innate (NKT cells) and adaptive systems), and dendritic cells (DCs) [42], which exert a net immunosuppressive function [50]. This prevents excessive inflammation in response to bacterial components and dietary antigens coming from the gastrointestinal tract through the portal vein. In addition, liver immune cells and inflammatory signals, such as IL-1β, IL-6 and TNF-α are necessary for liver regeneration in response to liver damage (Figure 1).

In CLD, the immune system is dysregulated by an excessive production of pro-inflammatory cytokines (IL-1β, IL-6, TNF-α), induced by the release of damage-associated molecular patterns (DAMPs) by dying hepatocytes [50]. Initially, KCs kill damaged hepatocytes and tumor cells via phagocytosis and secrete the aforementioned pro-inflammatory cytokines and chemokines (e.g., CCL2) to recruit and activate other immune cells. All this leads to the recruitment of monocytes (that differentiate into macrophages), neutrophils and other immune cells (DCs and CD8+ T cells), which amplify the response [51]. The interaction between platelets and KCs also induces the secretion of KC-derived cytokines and chemokines that promote NK and CD8+ T cell recruitment, at least, in NASH [52,53]. KCs also release TGF-β1 and PDGF, activating HSCs. NKs would kill activated HSCs in the early stages, preventing fibrosis progression [54,55]. However, differences in the response of KCs and other immune cells can take place during CLD and its progression to HCC depending on the etiology of the disease. For example, during NAFLD/MAFLD progression to HCC, resident KCs are quickly depleted and replaced by monocyte-derived KCs and hepatic lipid-associated macrophages [56], which can also derive from adipose tissue multipotent adult stem cells in obesity. Hence, serum levels of factors, like stem cell growth factor-beta (SCGF-β), that regulate macrophage differentiation might be useful in predicting liver disease severity and insulin resistance in obese patients, although further studies are required [57].

DCs play an important role both during CLD progression and once HCC is generated. They infiltrate the tumor, presenting antigens from tumor cells to T cells to prime and co-stimulate CD8+ T cells [43]. CD4+ T cells are also involved in the immune response in CLD-HCC with different populations of these cells exerting specific, or even opposed actions [52].

CD8+ cytotoxic T cells (CTLs) are the main killers of cancer cells upon recognition of antibodies on DCs [43]. CD4+ T cells can also activate CD8+ T cells by CD40L and enhance their proliferation and maturation into memory CD8+ T cells in the initial tumor stages. Hence, increased infiltration of CD8+ T cells in HCC patients is associated with a better prognosis [50]. However, a high expression of regulatory factors such as VEGF, CXCL17 or IL-10 can restrict CTLs response. On the other hand, different subpopulations of CD8+ T cells can play specific roles depending on the context. Thus, using different NASH mouse models and experimental approaches, a pro-inflammatory and pro-tumorigenic role has been demonstrated for CD8+ T cells, facilitated by platelet-mediated recruitment [53,58]. Particularly, CD8+CXCR6+PD1+ T cells mediate the auto-aggressive killing of liver cells and are associated with chronic liver damage and HCC development in NASH [59,60]. Therefore, further understanding of their role in HCC is required, as it likely depends on the specific context and/or stage.

NK cells are essential to kill tumor cells through perforin and granulin release. In addition, they produce pro-inflammatory cytokines and chemokines that contribute to their anti-tumor function [43]. However, liver specific NK cells are maintained in a hyporesponsive state, even after their activation by pro-inflammatory signals [61]. Moreover, the NK cell population decreases in blood and tumor tissues from HCC patients [50].

In response to liver damage, and in HCC, bone marrow derived monocytes are also recruited to the liver, where they differentiate into macrophages [62,63,64]. CCL2 secreted by hepatocytes, KCs, HSCs, and platelets [51] and acting through CCR2, is essential for this recruitment [65]. These recruited macrophages together with KCs constitute the tumor-associated macrophages (TAM) that accumulate within the tumor [42].

TAMs can undergo M1 (classic) or M2 (alternative) polarization, although an overlap in M1/M2 signatures also occurs. M1 macrophages are pro-inflammatory, hence they secrete TNF-α, IL-6 or IL-1β and produce reactive oxygen species (ROS) and nitric oxygen (NO) to eliminate tumor cells [66]. In contrast, M2 macrophages secrete anti-inflammatory cytokines (e.g., IL-10, TGF-β1), chemokines (e.g., CCL17, CCL22), and growth factors (e.g., VEGF, fibroblast growth factor (FGF)), which promote cancer progression, EMT, immune evasion and angiogenesis [42,67]. M2 TAMs replace M1 TAMs in the advanced HCC stages, Wnt ligands produced by hepatic tumor cells being important for M2 polarization [68].

Tregs are a subtype of CD4+ T cells that express CD25 and FOXP3 (forkhead box P3) [69]. Their main function is to maintain autoimmune tolerance and homeostasis by reducing the immune response. Consequently, once Tregs are recruited by CCL20/CCR6 [70] and activated by IL-10 and TGF-β1, they play an anti-inflammatory and immunosuppressive role in HCC through impairing CD8+ T cell actions [71]. Accordingly, high levels of Tregs are present in the TME of HCC patients with a poor prognosis [72,73]. However, it remains unclear how Tregs operate in certain scenarios, for instance, how they regulate NASH development and progression to HCC [52].

Myeloid-derived suppressor cells (MDSCs) are a heterogeneous population of bone marrow-derived immature myeloid cells, classified into granulocytes (also called polymorphonuclear (PMN-MDSCs)) and mononuclear (M-MDSCs) [74]. Their major role is to suppress anti-tumor immunity through the inhibition of DCs, CTLs, NK and B cells and to activate Tregs and TAMs, as well as to promote angiogenesis [43]. Their recruitment to the liver during HCC development and progression is mainly promoted by cytokines (e.g., granulocyte-macrophage colony-stimulating factor (GM-CSF), granulocyte colony-stimulating factor (G-CSF), and IL-6) and chemokines, such as CCL2, according to data derived from different murine models of HCC [75]. However, other signals like CCL26 could also contribute to it [42].

In HCC patients, MDSCs are increased in the blood and within the tumor, inducing programmed death-ligand 1 (PD-L1) expression in advanced stages through interaction with KCs [76] to suppress the adaptive immune response.

Tumor associated neutrophils (TANs) have a similar origin to that of PMN-MDSCs and share some phenotypic traits [77]. However, they are more heterogeneous and present different functional properties. Interferon β (IFN-β) induces an antitumor N1 polarization of TANs, which can kill tumor cells and activate CTLs. In contrast, the N2 phenotype is induced by TGF-β, cytokines and chemokines, promoting tumor development and immunosuppression through the inhibition of T-cell response, also enhancing angiogenesis [78,79]. N2 TANs produce CCL2 and CCL17, which promote macrophage and Tregs’ recruitment [80].

The recruitment of neutrophils into HCC tumor tissue, after G-CSF and IL-17-induced mobilization, is mediated by chemokines such as CXCL1–3 and CXCL5–8, which bind to the CXCR2 receptor in neutrophils [79,81]. Hence, CXCL5 secreted by tumor cells increases TANs infiltration, leading to a positive feedback loop [82].

TANs induce tumor cell migration and invasion and promote metastasis generation [79]. Neutrophils also protect tumor cells from being killed by NK cells [83]. Moreover, the formation of platelet-neutrophil clusters induced by platelet-derived CXCL5 and CXCL7 leads to the generation of premetastatic niches [84,85].

On the other hand, neutrophil extracellular traps (NETs) derived from TANs have emerged as important regulators of HCC [86]. NETs are released into the tumor tissue and circulation, and are able to trap tumor cells, which favors its spreading through the blood to generate metastasis [87].

More recently, B cells have been considered to play a role in HCC [1]. Studies derived from mouse models found both tumor-promoting and suppressing activities for B cells in HCC [88]. The release of selective cytokines by different populations of B cells could account for distinct effects in specific contexts [43].

### 2.4. Platelets

Platelets are also a component of the liver microenvironment and are key players in fibrosis, CLD and HCC. Under physiological conditions, platelets promote hemostasis and vascular integrity, also regulating the immune system, which allows for the protection of the liver from pathogens [89]. In CLD and HCC their function seems to be dual (Figure 1). Hence, both protective and deleterious effects of platelets have been described, playing specific roles in different pathologies and pathological stages [89].

Different clinical studies have shown that the presence of thrombocytosis is associated with worse prognosis in patients with HCC [90], unlike thrombocytopenia, which promotes patients’ survival [91]. Thus, a high platelet count directly correlates with greater tumor aggressiveness and poorer survival of HCC patients [91]. In addition, clinical trials have shown that antiplatelet therapy using clopidogrel or aspirin reduces liver fibrosis risk [92] and HCC, and decreases liver-related mortality in patients with chronic hepatitis B and C. This suggests that platelets may contribute to HCC progression [93,94,95]. Moreover, patients with advanced HCV-induced fibrosis or NASH had increased serum and intrahepatic levels of platelet-derived CXCL4, which promotes HSC proliferation, chemotaxis, and chemokine expression [96]. Therefore, platelet count has been incorporated as a prognostic marker, and a criteria for the selection of treatment in patients at risk of developing HCC and in patients with viral liver cirrhosis [97,98,99].

In HCC, platelets interact with different liver cells, including HSCs, LSECs and immune cells, in addition to having a potential direct effect on tumor cells [100,101]. In mouse models of acute viral hepatitis, platelet depletion decreases intrahepatic accumulation of virus-specific CTLs and delays organ damage, suggesting that platelets can enhance adaptive immunity [102]. In agreement with this, in an HBV transgenic mouse model, antiplatelet therapy inhibits liver fibrosis and HCC development by reducing the number of intrahepatic HBV-specific CD8+ T and virus-non-specific inflammatory cells [103].

In other types of liver injury, platelets also promote damage, fibrosis and HCC. For instance, platelet number, activation and aggregation are increased in NASH [53]. In the early and advanced stages of NAFLD, platelet recruitment to the liver is mediated by KCs through hyaluronan-CD44 binding, and platelet activation by Glycoprotein GPIbα, contributing to NASH and HCC development [53]. Antiplatelet therapy with aspirin or clopidogrel blocks intrahepatic immune cell infiltration, reduces inflammatory cytokines and hepatocyte damage, preventing NASH-induced HCC [53]. Furthermore, in MDR2(Abcb4)-null mice, and control mice fed with a 3,5-diethoxycarbonyl-1,4-dihydrocollidine (DDC)-supplemented diet that leads to cholestasis, platelets activate HSCs through PDGF-β secretion and promote fibrosis [104]. In contrast, our unpublished results indicate that livers from transgenic mice overexpressing C3G in platelets are partially protected from carbon tetrachloride (CCl_4_)-induced fibrosis, while the deletion of platelet C3G increases liver fibrosis. This suggests that platelet C3G, which is involved in platelet activation [105,106], has a protective effect on fibrosis. However, diethylnitrosamine (DEN)+CCl_4_-induced liver cancer is exacerbated in wild type (wt) mice as compared to mice lacking platelet C3G, which suggests that platelet C3G promotes HCC progression. Therefore, these studies support the idea that platelets regulate liver immune response and can either promote or reduce fibrosis. However, in all these contexts platelet activation contributes to HCC development and/or progression in different scenarios of liver chronic damage.

Nevertheless, other studies have shown that platelets can also have a protective role in liver regeneration, CLDs and fibrosis, also preventing HCC. For instance, platelet-derived serotonin induces hepatocyte proliferation in a model of liver regeneration, and the release of growth factors and cytokines (e.g., IL-6), at the site of liver injury [107]. Thrombopoietin mediated platelet generation after liver hepatectomy also reduces liver fibrosis and promotes liver regeneration [108]. In a model of CCl_4_-induced fibrosis, platelets also reduce liver fibrosis progression through MMP-9 induction and TGF-β downregulation [109]. Human platelets also attenuate liver fibrosis in severe combined immunodeficiency (SCID) mice by secreting HGF that inhibits HSC activation, upregulates MMPs, and restrains hepatocyte apoptosis [110]. Furthermore, in NAFLD mouse models, platelets restrain HCC growth by enhancing CD8+ T cell-dependent anti-tumor immunity through P2Y12/leukotrienes-dependent CD40L release [111]. Moreover, in a mouse model of cholestasis-induced liver fibrosis, platelets are activated by HSCs and play an antifibrotic role by reducing collagen I deposition by HSCs through enhancing the HGF-MET signaling pathway [112].

Altogether, all these data support that platelets are relevant regulators of CLD and liver cancer through modulation of immune cells, HSCs and other liver cells. However, their effects are likely dependent on the context.

### 2.5. Hepatic Stem/Progenitor Cells in Liver Cancer

The adult hepatic stem/progenitor cell (HPC) population constitutes another cellular component of the liver microenvironment during chronic liver injury. Evidence in rodents and humans locates the liver stem cell niche at the Canals of Hering—the adult remnants of fetal/neonatal liver ductal plates—in the periportal area. From there, a transit amplifying cell population of bipotent cells, capable of differentiating into both hepatocytes and cholangiocytes arises and expands in a context of CLD to repair the liver [113,114,115]. This makes HPCs potential candidates as cells of origin in liver cancer, since chronic liver injury constitutes a predominant risk factor for all types of liver tumors (HCC, CCA, and cHCC-CCA) that precedes the big majority of them [116,117,118]. Nonetheless, the potential of HPCs to generate liver tumors has been a subject of intense debate. There is compelling evidence for HPCs’ tumorigenic potential (thoroughly discussed in other recent reviews) [119,120,121]. The strongest evidence is the fact that HPCs/oval cells are susceptible to neoplastic transformation, giving rise to HCC upon genetic alterations implying activation of oncogenes (such as *Ras*) or silencing of tumor suppressors (including *ARF/INK4a* and *p53*) [122,123,124,125]. In the same line, HCC tumors spontaneously developed in mice with a deletion in embryonic liver fodrin (*ELF*), an adaptor protein required for TGF-β signaling. These tumors were proposed to derive from transformed stem cells with the inactivation of TGF-β signaling and subsequent activation of IL-6 signaling [126]. In agreement with this, chronic exposure of HPCs to TGF-β can confer tumor initiating cell properties and promote hepatocarcinogenesis through a miR216a/Phosphatase and Tensin Homolog (PTEN)/Akt-dependent pathway, under specific conditions [127], but not all, based on our own data [128]. Furthermore, conditional deletion of mammalian orthologs of Hippo kinase, the serine-threonine kinases Mst1 and Mst2, leads to excessive proliferation and appearance of liver tumors at 5–6 months of age, preceded by accumulation of small proliferating periductal cells expressing HPC markers. This, together with the observation of tumors of both biliary and hepatocyte lineages, made authors link HPC activation by suppression of Hippo pathway with tumor formation [129]. These findings are in accordance with gene expression profiling data that identified a subset of HCCs consistent with a an HPC origin, which is among those with the worst prognosis [130,131]. Altogether, evidence supports that, at least, a fraction of HCC could derive from transformed HPCs as a consequence of the accumulation of genetic alterations and a cell maturation arrest process. Nevertheless, the key issue is which cell is more prone to neoplastic transformation, mature hepatocyte or HPC, and which of them more likely becomes a tumor-initiating cell. The use of state-of-the-art strategies is helping on this. One recently reported experimental model in this field is a double knockout mouse combining the loss of autophagy (*ATG5* or *7*) and *PTEN* genes that results in inflammation and fibrosis, concomitant with the development of an extensive ductular reaction, which leads to HCC formation at 4–5 months of age. Lineage tracing-based studies revealed that HCCs generated from ductular liver progenitor cells are derived from dedifferentiated hepatocytes. These findings prompt us to be more careful when interpreting the cellular origin of HCC based on the stem/progenitor-like properties of tumor cells, and support that hepatocytes are the cells of origin of HCC, at least, upon injury induced by autophagy deficiency [132]. Earlier lineage tracing studies in Rosa-YFP (yellow fluorescent protein) mouse reached similar conclusions. Using adeno-associated virus (AAV)-Thyroxine binding globulin (TBG)-Cre or Foxl1-Cre mice to label hepatocytes or stem/progenitor cells, respectively, and two models of chemical hepatocarcinogenesis (induced by DEN/CCl_4_ or DEN/TCPOBOP (1,4-Bis[2-(3,5-Dichloropyridyloxyl)] benzene), the authors also demonstrated that tumor formation was driven by transformed hepatocytes [133]. These studies highlight that oncogenic reprogramming and acquisition of stemness in hepatocytes can be important steps in HCC development. On the other hand, Tummala et al. [134] using a mouse model expressing hepatocyte-specific human unconventional prefoldin RPB5 interactor (hURI-tetOFFhep) that causes DNA damage and results in liver tumor development, demonstrated that both HPCs and adult hepatocytes can contribute to liver tumorigenesis. However, about 70% of HCCs originate from hepatocytes, regardless of HPC expansion during early stages of hepatocarcinogenesis. Of note, studies with Mdr2-KOFoxl1-Cre; RosaYFP mice that develop both HCC and cHCC-CCA tumors showed that HPCs are the source of cHCC-CCA tumors, but not of HCCs [133,135]. Hence, no universal conclusions should be made from specific experimental models. Clearly, different cancer models lead to different tumorigenic processes, making it clear that the cell of origin of HCC is context specific. Nonetheless, the idea of HPCs as a major source of liver tumors, specifically HCC, should be dismissed, since there is sufficient evidence supporting a relatively low risk of their malignant transformation. This is an interesting issue to keep in mind when evaluating therapeutic strategies to push regeneration in CLD.

## 3. Signaling Pathways in HCC

Among all signaling pathways regulating liver cancer development and progression, we have focused on those elicited by the receptor tyrosine kinases (RTKs) MET and Epidermal growth factor receptor (EGFR), and TGF-β.

### 3.1. Tyrosine Kinase Receptors: MET and EGFR

HGF acting through MET represents a key signaling pathway that controls epithelial morphogenesis through its mitogenic, motogenic and pro-survival activities, being essential for liver development [136,137,138]. HGF/MET signaling is also necessary for reparative responses, activated upon liver injury, both during hepatocyte-mediated regeneration and liver regeneration associated with HPC expansion [139,140,141,142] and exerts hepatoprotective effects against liver fibrosis through different mechanisms [143].

A number of in vitro and in vivo analyses have been performed to clarify the role of HGF/MET in HCC. In vitro data support the notion that HGF/MET has a pro-invasive effect and relate it to its ability to induce EMT [144,145]. Thus, HCC cell lines with high MET levels display a mesenchymal phenotype [146,147]. Different laboratories have analyzed the effects of inactivating or overexpressing MET and/or HGF in in vivo experimental models of HCC with discordant results. As an example of the complexity of MET signaling effects in HCC, the loss of MET in hepatocytes led to bigger tumors with shorter latency compared to controls in the DEN-induced HCC model. On the contrary, transgenic mouse models of MET overexpression in hepatocytes spontaneously generate liver tumors [148,149,150]. On the other hand, MET knockout accelerates and enhances chemically-mediated HCC initiation, but does not affect phenobarbital-induced HCC promotion [151]. Therefore, the scenario is intricate and suggests that context might be important, and a fine balance of HGF/MET signaling is necessary to maintain liver homeostasis.

It is well established that the HGF/MET signaling pathway is overactivated in around 50% of HCC patients and nearly all liver metastases. Furthermore, comparative functional genomics identified a MET-regulated signature in a subgroup of HCC with aggressive phenotype and poor prognosis [152]. Aberrant activation of MET signaling can be achieved by alterations at different levels, including gene amplification, activating mutations, downregulation of microRNAs targeting MET, and autocrine signaling due to HGF overexpression or activation by other ligands [153]. However, neither MET expression nor HGF plasma levels can be used as diagnostic or prognostic factors in HCC, as studies led to controversial results. Therefore, it deserves further analysis. One possibility is their potential utility in specific patient subgroups, particularly those with the MET specific signature, or pathological status. Another possibility would be to use them together with other HCC risk parameters [154,155].

Importantly, the HGF/MET axis has emerged as a therapeutic target in HCC. Different types of HGF inhibitors have been designed, including HGF neutralizing antibodies, HGF antagonists, MET-selective TKIs or multitarget TKIs [156]. Cabozantinib belongs to this last group, and it has recently been included as a second line targeted therapy. Cabozantinib targets MET and receptors associated with angiogenesis (such as VEGFR or AXL). How the effect on these different targets contribute to reaching the final antitumoral effect is not known, nor the specific contribution of MET inhibition to the overall effect. Data obtained with selective TKIs shed light on this question. Positive results obtained in clinical trials with Tepotinib and Capmatinib in MET positive-advanced HCC [157] support a key contribution of MET inhibition to the final effects of TKIs. However, HGF/MET targeting in HCC presents some key problems still unresolved. First, understanding why other TKI drugs, such as (ARQ 197), a TKI selective for MET [158] or the multitarget TKI, golvatinib, in combination with sorafenib [159], failed to be effective for advanced HCC treatment. Second, due to its important regenerative functions, the inhibition of the HGF/MET axis may be detrimental for the liver disease that frequently accompanies HCC development [156]. Targeting HGF/MET downstream effectors could solve some of these problems that current therapy, focused on the receptor level, presents. Thus, the identification of specific and druggable targets in the HGF/MET signaling pathway, rather than focusing on proteins shared with other pathways, is desirable. Third, sorafenib, an oral multikinase inhibitor, is one of the options for standard first-line systemic therapy for HCC [160]. However, patients acquire resistance within 6 months, which is one of the major challenges of HCC management [161]. As aberrant activation of HGF/MET is one of the mechanisms involved in sorafenib resistance in HCC [162], inhibition of this pathway could aid to obtain better results with the current therapy. However, it is also necessary to consider (as described in other sections) that HGF is produced by different components of the liver TME. In addition, HGF/MET signaling plays specific roles in different liver cells that change during liver tumor generation and progression. Therefore, this needs to be taken into consideration.

EGFR is another member of the RTK family, activated by several ligands including TGF-α, AREG and EGF [163,164]. Together with its well-established role in liver regeneration [165], EGFR signaling is also involved in different stages of CLD. Hence, numerous studies point to a pro-fibrogenic role of EGFR in the liver [166,167,168,169]. Various EGFR ligands are overexpressed in response to chronic liver injury as shown in experimental models and human cirrhotic tissue. This may favor the hepatocarcinogenic process, as observed in different models of hepatic injury ending in HCC, in which pharmacological or genetic inhibition of EGFR prevents tumor development [170,171,172]. It is interesting to point out that EGFR is expressed in liver macrophages both in human HCC and mouse HCC models, being critical for HCC development as demonstrated by specific deletion in KCs/macrophages [173]. The activation of EGFR has also been detected in liver macrophages in experimental models of chronic injury [168,174]. These findings, together with a delay in the DEN-induced inflammatory response and tumor formation in mice expressing a dominant negative mutant EGFR lacking catalytic activity in hepatocytes [172], evidence an interesting regulatory role for EGFR in the HCC inflammatory microenvironment, which involves both hepatocytes and immune cells, underneath its pro-tumorigenic effect. Additionally, EGFR-dependent activation of HSCs has also been reported [175], which provides a potential additional mechanism by which EGFR would contribute to fibrogenesis, the activation of inflammation, and therefore, liver tumor formation.

Although aberrant EGFR activation through EGFR gene amplification and/or mutation has been detected in various types of cancer, this is not frequently found in HCC. In fact, EGFR overexpression is often observed in HCC, but it is not usually associated with an increase in gene copy number [176,177,178] or somatic mutations in the exon encoding the catalytic domain [179]. Interestingly, regulators of EGFR signaling have been described as altered, e.g., ERBB receptor feedback inhibitor 1 (ERRFI1), a negative regulator of EGFR that inhibits its catalytic activity and mediates its lysosomal degradation, is frequently deleted in HCC (Cancer Genome Atlas Research Network, 2017) [180]. EGFR ligands are also overexpressed in HCC [178,181,182]. Importantly, EGF is part of several gene signatures of different nature that are associated with HCC (angiogenic-, immune- related, and others) [154,183]. This suggests that EGFR signaling contributes to HCC development at different mechanistic levels.

Other evidence supports a key role for EGFR signaling in different types of liver cancer. Thus, EGFR expression correlates with a high proliferative activity, the presence of intrahepatic metastasis, poor differentiation, and bad prognosis in HCC [177,184]. EGFR activation is also associated with poor prognosis in carcinomas of the biliary tract [185].

All this, together with encouraging in vitro data using different types of EGFR signaling inhibitors (TKIs and EGFR targeted antibodies), paved the way for clinical trials aiming to impact EGFR signaling in HCC. However, to date, EGFR inhibition therapy cannot be used for HCC treatment [182], as clinical trials were not successful (Table 1), even the combination of inhibitors of different signaling molecules. For example, the TKI erlotinib—a potent EGFR inhibitor—alone (trial 1) or in combination with bevacizumab (anti VEGF) (trial 2) were administered in three different phase II clinical trials (NCT00365391, NCT02273362 and NCT00356889, respectively) [186,187,188]. The two formers induce a poor partial response (PR) (1 patient out of 27), a median time to disease progression of 3.0 months and a median survival time of 9.5 months in the combined therapy [189]. The latter, evaluated in patients with CCA, showed a PR of 12% after 6 months, a survival time of 9.9 months and a progression free survival (PFS) of 4.4 months.

The combination of erlotinib plus sorafenib phase II (NCT01093222) [190] also failed due to a very low PFS (of 2 months) and an overall survival (OS) of 6 months [190]. The safety and efficacy of the TKI lapatinib were also studied in a phase II trial (NCT00107536). The results indicate it is safe, but its OS and PFS are 12.6 and 1.9 months, respectively. Hence, efficacy needs further investigations [191].

Panitumumab (a monoclonal anti-EGFR antibody) plus gemcitabine and irinotecan [192] have also been tested in a phase II, showing a response of 31.4% and good tolerability, but in a short period of 5 months.

Based on all this, further research is required to define the resistance mechanisms to EGFR inhibition that operates in HCC. In this sense, EGFR signaling as a driver or modulator of liver inflammation in CLD and cancer [193,194] should be taken into consideration when thinking about EGFR inhibition to combat HCC. A better comprehension of this specific aspect of the EGFR signaling role in liver disease deserves further research. In this regard, it would be important to better understand the specific actions of EGFR signaling in the liver TME.

### 3.2. TGF-β

It is widely recognized that TGF-β plays critical roles in tumor initiation, development, and the generation of metastasis in several cancer types. Strikingly, TGF-β switches from a potent cytostatic and pro-apoptotic effect in normal epithelial cells to a tumor promoter activity at the late stages of the disease, a phenomenon known as the “TGF-β paradox” [195]. In a revealing work by Coulouarn et al. (2018) [196] two different TGF-β gene signatures were proposed in HCC. An “early signature”, characterized by the expression of genes related to tumor suppressor activities, and associated with the longer survival of HCC patients, and a “late signature” that includes a set of genes related to TGF-β tumor promoter effects such as positive regulators of cell cycle and EMT.

TGF-β is involved in all stages of CLD, from steatosis and inflammation to fibrosis, cirrhosis and HCC [195,196,197,198]. Therefore, many clinical studies point at TGF-β as a strong candidate for being a diagnostic and prognostic marker, as well as a target of therapeutic strategies. In fact, circulating TGF-β1 levels are elevated in HCC patients [199], which likely makes it a suitable marker for HCC diagnosis. The expression of TGF-β1 is also significantly higher in HCC tissues than in normal liver, and a correlation between TGF-β levels and poor prognosis, extrahepatic metastasis, lower survival rates and lower post-operative disease-free survival has been established [200].

The idea of targeting TGF-β in HCC is certainly promising but not that simple. Unfortunately, how HCC cells surpass the suppressive effect of TGF-β is not completely clear, although a few mechanisms have been described. Inactivating somatic mutations is one of them. A recent genomic and transcriptomic study [201] reported that almost 40% of HCC samples contain mutations in genes of the TGF-β signaling pathway. Some of them correlate with the inactivation of the pathway, due to the loss of TGF-β suppressor activity, while others result in an overactivation of the pathway, again making evident the TGF-β paradox. Other mechanisms include alterations in TGF-β receptor internalization [202,203]; epigenetic silencing events [204]; aberrant epitranscriptomic RNA modifications [205] or the expression of specific miRNAs that allow cells to escape from TGF-β-induced apoptosis [206]. Additionally, the overactivation of survival pathways that interfere with the Smad canonical pathway and/or crosstalk with non-canonical TGF-β signaling impacting on transcriptional regulation and the suppressive effects of TGF-β can also occur [207,208,209].

Likewise, the pro-tumorigenic effect of the TGF-β pathway is mediated by complex and diverse mechanisms that combine actions on the cancer cell itself, but also on the tumor stroma and microenvironment [210]. In this respect, since TGF-β is already present during the inflammatory response activated in CLD, it could build up a microenvironment optimal for HCC growth. TGF-β is an important player in the dialogue between cancer and stroma cells, as mentioned before, favoring the secretion of cytokines, like CLCF1 (CAF-derived cardiotrophin-like cytokine factor 1) [211]. This effect, together with the fact that TGF-β is a potent inducer of EMT, result in a positive feedback loop that sustains HCC cells´ proliferation and invasion through the release of growth factors, chemokines and cytokines. TGF-β is also a pro-angiogenic factor in HCC, acting both directly on the endothelial cells or indirectly, modulating the production of VEGF [212,213]. Finally, TGF-β is a key immunoregulator with immunosuppressive effects on innate and adaptive immune cells that result in tumor immune escape in HCC [214].

Encouraged by the promising results of clinical trials of galunisertib (a TGF-βRI kinase inhibitor factor), alone or in combination, for the treatment of advanced HCC [215], three different phase II trials (Table 1) investigated galunisertib, alone or in combination with sorafenib (NCT02178358) [216], the anti-PD-1 antibody nivolumab (NCT02423343) [217] or the anti-VEGFR antibody ramucirumab (NCT01246986) [218]. In the first one, neither the single nor the combination approach showed any benefit in OS nor in PFS. The second one had only one patient, and therefore, no conclusions can be drawn. For the latter, the combination with ramucirumab lacked results, but the combination with nivolumab showed better PFS. Both approaches showed similar reduction of AFP (alpha-fetoprotein) and TGF-β levels after the treatments [219]. Therefore, anti TGF-β therapy needs to be reevaluated considering the complexity of TGF-β effects. On one hand, further research is required to clarify the mechanisms behind the “TGF-β paradox” in HCC and to better understand the diverse functions of TGF-β in the different hepatic cell populations, and its secretion by several liver cells and platelets. On the other hand, a careful stratification of patients according to their TGF-β profile may help to guide clinical decision to improve patient outcomes.

**Table 1:** Completed clinical trials for HCC or CCA with published results.

Trial ID	Study Title	Target	Intervention	Results	Phase	References
NCT01271504	E7050 in combination with Sorafenib versus Sorafenib alone as first line therapy in participants with hepatocellular carcinoma (HCC)	MET	Golvatinib Sorafenib	- TEAEs: 100% - SAEs: 47.6% - TTP: 10.29 weeks - PFS: 10.29 weeks - OS: 27.86 weeks - OR: 4.8%	1–2	[159]
NCT00107536	Lapatinib ditosylate in treating patients with unresectable liver or biliary tract cancer	EGFR	Lapatinib	- PFS: 1.9 months - OS: 12.6 months - Target-EGFR/EGFR-P protein expression: 0% - Expression profile and mutations of genes critical for EGFR and ERBB2 Signaling: 0%	2	[191,220]
NCT00365391	Bevacizumab and Erlotinib in treating patients with advanced liver cancer	EGFR	Erlotinib Bevacizumab	- OR: 4.3% - TTP: 3 months - OS: 9.5 months - TTF: 2 months	2	[187]
NCT00356889	Bevacizumab and Erlotinib hydrochloride in treating patients with metastatic or unresectable biliary tumors	EGFR	Erlotinib Bevacizumab	- OR: 12% - OS: 9.9 months - PFS: 4.4 months - DOR: 8.4 months	2	[186]
NCT00753675	Vandetanib, Gemcitabine or placebo plus Gemcitabine or Vandetanib monotherapy in advanced biliary tract cancer	EGFR	Vandetanib Gemcitabine	- PFS: 105–148 days - OR: 3.5–15.5% - DOR: 127–277 days - OS: 228–307 days	2	[221]
NCT00948935	Study of Gemcitabine, Irinotecan and Panitumumab in patients with advanced and metastatic biliary tract adenocarcinoma	EGFR	Panitumumab Gemcitabine Irinotecan	- PFS: 69% - OR: 31.4%	2	[192,222]
NCT01093222	Sorafenib Tosylate and Erlotinib hydrochloride in treating patients with locally advanced, unresectable, or metastatic gallbladder cancer or cholangiocarcinoma (CCA)	EGFR	Erlotinib Sorafenib	- PFS: 2 months - OS: 6 months - OR: 6% - TEAEs: 100%	2	[190]
NCT02273362	Erlotinib hydrochloride in preventing liver cancer in patients with cirrhosis of the liver	EGFR	Erlotinib	- Response (at Least a 50% Reduction in Liver Phospho-EGFR Staining): 100, 60, 60% - TEAEs: 0–16.7%	1–2	[188,189]
NCT01246986	A Study of LY2157299 in participants with HCC	TGF-β	Galunisertib Sorafenib Ramucirumab	- Change from baseline in relationship of AFP to OS: 17.9–24.2% - Change from baseline in relationship of TGF-β to OS: 10.1–22.88% - TTP: 7.1–36 weeks - OS: 29.6–89.6 weeks - PFS: 6.6–28.4 weeks - OR: 0.0–3.7% - DOR: 37.6–47.2% - TTF: 9.9–49.3 weeks - TTW: 113–113 days	2	[218,219,223]
NCT02178358	A Study of LY2157299 in participants with advanced HCC	TGF-β	Galunisertib Sorafenib	- OS: 75–81.6% - TTP: 1.4–4.1 months - PFS: 1.4–4.1 months - OR: 0.03–0.16%	2	[216]
NCT02423343	A Study of Galunisertib (LY2157299) in combination with Nivolumab in advanced refractory solid tumors and in recurrent or refractory NSCLC, or HCC	TGF-β	Galunisertib Nivolumab	- PFS: 5.26, 5.39, - OR: 24, 0% - DOR: 9.03 months - TTR: 4.2 months - OS: 11.99–14.52 months	1–2	[217]

Compilation of the interventional clinical trials targeting MET, EGFR, or TGF-β, registered at the EU Clinical Trials Register (https://www.clinicaltrialsregister.eu, accessed on 28 September 2023) and the U.S. National Library of Medicine (https://clinicaltrials.gov, accessed on 28 September 2023). Only completed interventional trials with results posted are listed. Abbreviations: (TEAEs) Treatment-emergent Adverse Events; (SAEs) Serious Adverse Events; (TTP) Time to Progression; (PFS) Progression Free Survival; (OS) Overall Survival; (OR) Overall/Objective response (Complete Response or Partial Response); (DOR) Duration of Tumor Response; (TTF) Time to Treatment Failure; (TTW) Time to Worsening of Symptoms. Glossary: (TEAEs) Treatment-emergent Adverse Events. Undesirable events not existing prior to medical treatment, or an already existing event that worsens either in intensity or frequency following the treatment. (SAEs) Serious Adverse Events. A life-threatening adverse event (related with the medical treatment). (TTP) Time to Progression. The interval of time from the start of treatment to disease progression. Similar to (TTW) Time to Worsening of Symptoms. (PFS) Progression Free Survival. In a clinical trial, the interval of time from random assignment (placebo or treatment) to disease progression or death from any cause. (OS) Overall Survival. In a clinical trial, the length of time from randomization to death (OR) Overall/Objective response (Complete Response or Partial Response). The percentage of patients who achieve a complete response or partial response. Complete response: total disappearance of lesions. Partial response: reduction in the sum of maximal tumor diameters by at least 30% or more (following Response Evaluation Criteria in Solid Tumors (RECIST)). (DOR) Duration of Tumor Response (also known as Duration of clinical benefit (DoCB)). In a clinical trial, the period from randomization to disease progression or death in patients who achieve complete or partial response. (TTF) Time to Treatment Failure. In a clinical trial, the interval from chemotherapy (treatment) initiation to premature discontinuation (loss of efficacy, SAEs, death, patient voluntary termination, etc.).

### 3.3. C3G, a New Signaling Player in HCC

C3G (Crk SH3-domain-binding guanine-nucleotide-releasing factor), encoded by *RapGEF1* gene is a guanine nucleotide exchange factor (GEF) for Rap and other Ras proteins [224], which can also act through mechanisms not dependent on its GEF activity [225]. C3G plays a dual role in cancer acting as either a tumor suppressor or promoter depending on tumor type and stage [225,226,227,228,229].

**Figure 2:**
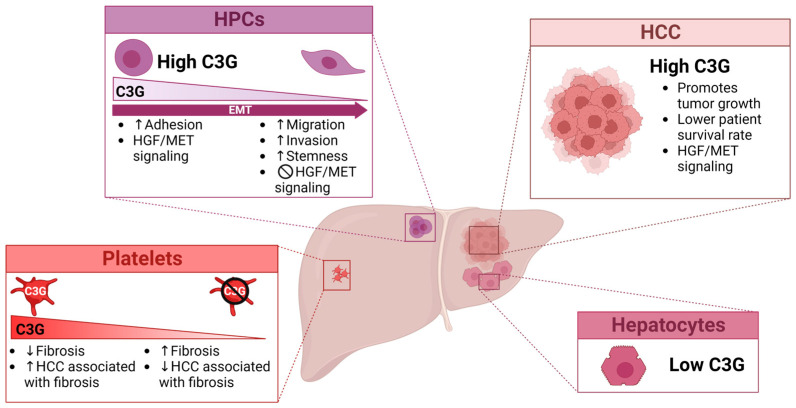
Functions of C3G in liver cancer. The scheme shows C3G functions in liver cancer at different levels. C3G protein expression is low in adult hepatocytes but increases in HCC cells promoting tumor growth and HGF/MET signaling. High C3G levels in patient tumor samples are associated with poor survival in patients. C3G present in platelets also contributes to enhance liver tumor growth in a mouse model of HCC associated with fibrosis (our unpublished data). On the other hand, hepatic progenitor cells (HPCs) express high C3G levels, which facilitate adhesion and allow a proper HGF/MET signaling. In contrast, low levels of C3G increase cell migration, invasion and stemness in HPCs. Arrows indicate upregulation (↑) or downregulation (↓). Created in BioRender.com.

C3G is expressed at low levels in the adult liver and hepatocytes, while in HPCs and neonatal hepatocytes is highly expressed [227]. Importantly, C3G expression is upregulated in HCC patient samples, liver cancer mouse models and HCC cell lines, promoting tumor growth (Figure 2). Hence, high *RapGEF1* mRNA levels correlate with tumor progression and a lower patient survival rate. In addition to C3G upregulation, genomic databases analyses show that *RapGEF1* is commonly altered in liver cancer (HCC and CCA). An analysis performed on 1829 samples/1710 patients showed genetic alterations in 2% of the samples, amplification or missense events being the most predominant aberrations [230,231,232,233]. Interestingly, most missense mutations are located in conserved regions (encoding catalytic, N-terminal inhibitory, or proline-rich domains) and might have functional consequences dysregulating C3G activity. It is also important to highlight that C3G is necessary for a proper activation of HGF/MET signaling in HCC cells. Additionally, as mentioned before, our own unpublished data also suggest that platelet C3G favors DEN+CCl_4_-induced liver cancer.

On the other hand, recent published data suggest that the Crk-C3G-Rap1 pathway could improve endothelial cell integrity in HCC upon treatment with the anti-tumor compound lenvatinib, facilitating tumor suppression when combined with anti-PD-1 [234].

C3G also regulates HPC biology [235]. C3G down-regulation favors a partial EMT associated with stemness and enhanced migration. C3G is required for HGF/MET signaling and its pro-invasive activity in HPCs, while TGF-β signaling is enhanced when C3G is down-regulated (Figure 2). Therefore, changes in C3G levels alter HPC signaling, which may impact liver repair in CLD. Thus, C3G down-regulation is detected in livers from DDC-treated mice, an experimental model of cholestatic liver injury in which HPC expansion occurs. Whether this might become relevant for HPC malignant transformation and HCC development in specific contexts deserves further analysis.

## 4. Liver Cancer Mouse Models

Several mouse models have been used to study liver cancer development, including genetically engineered mouse models, exposure to chemical agents, intrahepatic or intrasplenic injection of tumor cells and xenograft approaches [236]. The choice of the appropriate model depends on the scope of the research since these models cover many conditions that go from the chronic administration of chemicals to induce tumor development to acute injection of tumor cells (HCC or CCA). Additionally, these methods can be combined with extra agents to mimic the liver disease environment using specific diets, injection of chemotoxic agents or the expression of inflammation promoting genes. We will briefly review the available animal models on liver cancer with special focus on the pathways mentioned previously.

Several genetically engineered mouse models mimic HCC/CCA based on the activation of oncogenes or the inactivation of tumor suppressor genes through different strategies that go from genome editing to infection with HBV or HCV. Most of these strategies target tumor-related genes/pathways that have proven to be relevant in liver cancer like the MET/HGF axis, the EGFR, ErbB-2A, Wnt, P53, PTEN, Akt, TGF-α, Myc, E2F, or KRAS pathways (Table 2) [149,150,237,238,239,240,241,242,243,244,245,246,247,248].

Induced models include the use of agents (specific diets, carcinogens, etc.) to generate liver tumors [236,249,250]. Several chemotoxic agents have been used to promote liver cancer including agents able to alkylate DNA and/or promote oxidative stress (DEN, N-Nitrosomorpholine (NMOR), Dimethylnitrosoamine (DMN), 2-acetylaminofluorene (2-AAF), thioacetamide (TAA) [251,252], agents that promote *RAS* mutations (DMBA (dimethylbenz(a)anthracene) [253], or the cholangiocarcinogen furan [254,255,256,257]. Furthermore, dietary-based models have been developed to induce NAFLD/NASH or other pre-cancer conditions relying on either high content of nutrients (carbohydrate, fat and cholesterol) or nutrient deficient diets (Table 2) [258]. Among the second group the most frequently used are the methionine and choline-deficient (MCD), and choline-deficient, L-amino acid-defined (CDAA) diets [259,260,261,262,263,264]. These models of NASH may progress to HCC after 20 months. However, the natural resistance of mice to developing HCC makes them long-term models and sometimes unsuitable for liver cancer studies unless combined with other strategies. Thus, combined models have been used to promote HCC like the choline-deficient high-fat diet (CD-HFD) that combines obesity and NASH, promoting HCC development within 12–24 months. Similarly, choline-deficient, L-amino acid-defined, high-fat diet (CDA-HFD) induces NASH/NAFLD with a quick progression to HCC [258,259,263,264].

In addition to the previous models, the most common method to establish HCC or CCA is through the injection of tumor cell lines (implantation models) through heterotopic/orthotopic injections in immunodeficient mice models [236,265]. Additionally, the syngeneic models allow for the use of a recipient with a functional immune system granting the study of immune cells on tumor formation and progression. Lately, the implantation models have incorporated human immune cells that invade the tumor and mimic a more realistic TME (humanized mouse models). To fully recapitulate human disease HCC or CCA, liver cancer-induced models are usually combined with agents that are able to induce liver diseases (HBV, HCV, alcohol, CCl_4_ or special diet) [236,265].

Lastly, treatment to induce chronic cholestasis in combination with DMN or DEN promotes CCA and induces genetic aberrations that serve as the basis for several animal models of CCA [250,266].

Despite the great variety of models, most of them share common genetic alterations that are able to induce human liver cancer, some of the most relevant: oncogenes (*MYC*, *MET*, *RAS*, *NICD1*, *ERB2*, *NOTCH*, *AKT*), tumor suppressors (*P53*, *PTEN*, *FBXW7*), and the WNT pathway (*CTNNB1*) [236]. Several of these pathways converge on the activation of intracellular pathways that rely on small GTPases (Ras, Rac, Rho) to transduce the oncogenic signaling responsible for the cellular transformation highlighting the role of their modulators like GEFs and GAPs.

**Table 2:** Liver cancer mouse models.

**Genetically engineered mouse models**			
**Model (Oncogene/TSG)**	**Altered Pathway**	**Type of Cancer**	**References**
HBV, HCV	Viral model	HCC 13–24 months	[267,268]
WNT1, CTNNB1	Wnt pathway	HCC	[240,248]
NOTCH1	Notch pathway	CCA	[247]
P53, myc, E2F	Cell cycle	HCC	[237,245,246]
PTEN, PTEN/SMAD4	PI3K/Akt pathway	HCC, CCA (SMAD4/PTEN)	[243,244]
IGF2	Insulin growth factor pathway	HCC	[242]
EGFR, ERBB2	EGF pathway	HCC, CCA	[172,241]
HGFR (met), HGF	HGF signaling	HCC (combination with DEN, b-catenin)	[149,150,239,240,269,270]
TGF-α (+Myc), TGF-α/TGF-β	EGFR signaling	HCC	[271,272,273,274]
KRAS/HRAS	Ras signaling	HCC, CCA (combination with PTEN)	[237]
**Chemotoxic agents**			
**Model (agent)**	**Mechanism of action**	**Type of cancer**	**References**
DEN/DEN-CCL_4_	Genotoxic hepatocarcinogen	50–90 weeks 100% HCC	[252,257,275,276,277,278]
NMOR	Genotoxic	12 weeks HCC with lung metastasis	[279]
DMN	Alkylate DNA and/or promote oxidative stress	Promote HCC	[280,281]
2-AAF	Alkylate DNA and/or promote oxidative stress	Promote HCC	[282]
DMBA	Induces Ras mutation	Promote HCC	[253]
TAA	Genotoxic	Promote HCC and CCA	[251,252]
Furan	Genotoxic	Promote CCA	[256]
**Dietary models**			
**Model (diet)**	**Mechanism of action**	**Type of cancer**	**References**
High nutrient	NASH/NAFLD	more than 80 weeks/20% HCC	[258]
MCD	Oxidative DNA damage and chromosomal instability	30–35 weeks 25–100% HCC	[261,262]
CDE	Oxidative DNA damage and chromosomal instability	30–35 weeks 25–100% HCC	[260]
CDAA	Oxidative DNA damage and chromosomal instability	84 weeks 100% HCC	[259]
CDHFD	Oxidative DNA damage and chromosomal instability	30–35 weeks 100% HCC	[258,264]
CDAHFD	Oxidative DNA damage and chromosomal instability	30–35 weeks 100% HCC	[258,263]
**Implantation models** [264,265,266]			
**Model**	**Comments**	**Advantages**	**Disadvantages**
Heterotopic	Subcutaneous inoculation of human cultured cells	Quick evaluation of tumor growth	No immune response
Orthotopic	Liver implantation of human cultured cells	Reproduce TME in immunodeficient mouse	Unable to trigger an immune response
Syngeneic	Heterotopic or orthotopic implantation of mouse tumor cells	Reproduce TME and mimic the metastatic behavior of HC in immunocompetent mouse	Differences among human and mouse disease
Humanized mouse models	Transplantation of cancer patient tissue directly into immunodeficient mice	Genetic and histological similarities. Identification of treatments	Reflect human disease and allow pharmacological testing

Summary of the most relevant methods to induce liver cancer. Abbreviations: HCC, hepatocellular carcinoma; CCA, cholangiocarcinoma; DEN, diethylnitrosamine; CCL4, Carbon Tetrachloride; NMOR, N-Nitrosomorpholine; DMN, dimethylnitrosamine; 2-AAF, 2-acetylaminofluorene; DMBA, 9,10-dimethyl-1,2-benzanthracene; TAA, Thioacetamide; MCD, methionine and choline- deficient; CDE, choline-deficient and methionine-supplemented; CDAA, choline-deficient l-amino-defined diet; CDHFD, choline-deficient high-fat diet; CDAHFD, choline-deficient, L-amino acid-defined, high-fat diet.

## 5. Conclusions and Future Directions

Most recent studies reinforce the idea that the TME plays a key role in liver cancer, with platelets emerging as important players with a potential value for diagnosis, prognosis, and treatment. On the other hand, although fibrosis has been classically associated with HCC development and progression, several exceptions came out, as well as new mechanisms controlling fibrosis. Therefore, the complex interplay between fibrosis and liver cancer deserves a deep analysis to find out the mechanisms determining its positive or negative role. For this, it could be helpful to understand particular features (etiology- dependent and-independent) of the fibrotic process that define its specific impact on liver cancer.

It is also remarkable that HGF/MET/C3G, EGFR and TGF-β signaling regulates not only hepatocyte cell fate and function, but also the function of most liver cells including HSCs, CAFs, and immune cells. Moreover, HGF, EGF and TGF-β can be secreted by platelets and different cells from the liver microenvironment. This, together with the generation of resistance to treatments, explain the limited effects of HCC therapy with inhibitors of these pathways. Hence, new approaches should be considered to improve treatment based on patient classification into subgroups according to gene signatures that reflect not only tumor cell gene profile, but also tumor microenvironment and tumor stage.
